# Dry Water as a Promoter for Gas Hydrate Formation: A Review

**DOI:** 10.3390/molecules28093731

**Published:** 2023-04-26

**Authors:** Yu Wei, Nobuo Maeda

**Affiliations:** Department of Civil and Environmental Engineering, University of Alberta, Edmonton, AB T6G 2E1, Canada; ywei8@ualberta.ca

**Keywords:** gas hydrate, dry water, water-in-air dispersion, thermodynamic hydrate promoter, kinetic hydrate promoter, gas storage capacity

## Abstract

Applications of clathrate hydrate require fast formation kinetics of it, which is the long-standing technological bottleneck due to mass transfer and heat transfer limitations. Although several methods, such as surfactants and mechanical stirring, have been employed to accelerate gas hydrate formation, the problems they bring are not negligible. Recently, a new water-in-air dispersion stabilized by hydrophobic nanosilica, dry water, has been used as an effective promoter for hydrate formation. In this review, we summarize the preparation procedure of dry water and factors affecting the physical properties of dry water dispersion. The effect of dry water dispersion on gas hydrate formation is discussed from the thermodynamic and kinetic points of view. Dry water dispersion shifts the gas hydrate phase boundary to milder conditions. Dry water increases the gas hydrate formation rate and improves gas storage capacity by enhancing water-guest gas contact. The performance comparison and synergy of dry water with other common hydrate promoters are also summarized. The self-preservation effect of dry water hydrate was investigated. Despite the prominent effect of dry water in promoting gas hydrate formation, its reusability problem still remains to be solved. We present and compare several methods to improve its reusability. Finally, we propose knowledge gaps in dry water hydrate research and future research directions.

## 1. Introduction

Gas hydrate, also known as clathrate hydrate, is a compound with an ice-like structure in which guest molecules are trapped inside cages formed by hydrogen bonding between water molecules. Despite its similarity to ice, clathrate hydrate can remain stable above the melting point of ice because of the cages stabilized by the guest molecules. Many gas molecules with a low solubility in water, such as low-molecular-weight hydrocarbons, carbon dioxide, hydrogen, and nitrogen, can form hydrate crystals with water molecules when temperature and pressure conditions fall within the stable zone of the clathrate hydrate phase diagram. Compared to aqueous solutions, the amount of gas that can be accommodated in the form of clathrate is much greater, making it a promising option for gas storage applications. For example, CO_2_ hydrate can be used to capture and sequester CO_2_, which is a main greenhouse gas whose emission target was set to its reduction by about 45% by 2030 [1]. Transporting flammable or potentially explosive gases like H_2_ and natural gas is also safer with clathrate hydrate than with other methods like liquefaction. In contrast to guest gases, electrolytes are soluble in water and insoluble in clathrates, making clathrate hydrate useful for seawater desalination. Additionally, the differences in the thermodynamic conditions of different gas hydrate formations can be used for gas separation.

Though promising, the commercialization of clathrate hydrate applications is limited by the low water-to-hydrate conversion rate and slow kinetics, which are caused by mass transfer limitations [2] and heat transfer limitations [3]. Because most guest molecules have low solubility in water, the mass transfer limitation results in the highest supersaturation at the guest-water interface. When hydrate forms, it blocks further guest diffusion to the bulk water phase because the hydrate phase is mostly impermeable to gases, limiting clathrate hydrate formation to the interface where the guest gas contacts the water directly. Furthermore, the exothermic nature of clathrate hydrate formation lowers the driving force, thereby slowing down the nucleation kinetics.

To address the slow kinetics of clathrate hydrate formation, several methods have been employed. One such method is mechanical stirring, which has been shown to improve mass transfer, dissipate the latent heat released by hydrate formation, and increase the rate of hydrate formation [4]. Another method is the use of a surfactant, which was proved to be an effective kinetic hydrate promoter (KHP) by increasing the solubility of the guest in water and lowering the guest aqueous-phase interfacial tension [5,6,7,8,9,10,11]. The most commonly used surfactant has been sodium dodecyl sulfate (SDS) [12,13]. However, many surfactants are toxic and not suitable for some clathrate hydrate applications like water desalination or food processing [14]. Another challenge of using a surfactant as a KHP is the severe formation of foams, which have a long lifetime that persists long after hydrate dissociation [15]. To this end, amino acids were investigated and found to enhance the clathrate hydrate formation rate without raising the problem of toxicity and bubbling [16,17], thus being more environmentally friendly. Like surfactants, an amino acid is an amphiphilic molecule with a hydrophobic chain and a hydrophilic carboxylic head. The mechanism of amino acids of promoting hydrate formation is similar to that of surfactants [17]. However, the effectiveness of an amino acid in promoting hydrate formation kinetics was found to be dependent on the type of the guest gases [18] and the hydrophobicity of the amino acid [19]. Additionally, amino acid-based compounds [20] were reported to have a promoting effect on gas hydrate formation. Ionic liquids (ILs), which are a type of organic salt, have been found to be effective in promoting gas hydrate formation. Several studies have reported that certain ILs, such as 1-hydroxyethyl-1-methylmorpholinium chloride [21], aromatic-based ILs [22], choline octanoate [23], and 1-ethyl-3-methylimidazolium hydrogen sulfate [24], promote methane hydrate formation. Interestingly, the promoting mechanism of ILs differs from that of surfactants. However, it is worth noting that both amino acids and ILs can either promote or inhibit hydrate formation, depending on their composition.

Other environmentally friendly gas hydrate promoters have been developed to comply with increasingly strict environmental regulations, in addition to amino acids. Ignosulfonates [25,26,27] have been reported to promote gas hydrate formation due to their capillarity-driven characteristics, which improve gas diffusion from the bulk guest gas phase into the aqueous phase [28]. Biosurfactants, such as surfactin [29] and rhamnolipid [30], have the same mechanism as synthetic surfactants in promoting gas hydrate formation, but are more biodegradable.

Recently, hydrate seed solutions have been utilized as nucleation sites to facilitate hydrate formation [31,32,33]. This technique involves the use of pre-formed gas hydrate crystals, such as cyclopentane hydrate, under mild conditions as hydrate seeds to promote gas hydrate formation that would otherwise require more demanding conditions, such as hydrogen hydrate and methane hydrate. Experimental results have confirmed its effectiveness in accelerating gas uptake and promoting the formation of hydrocarbon-hydrogen mixed gas hydrates.

Non-chemical-based methods have also been employed to enhance the kinetics of hydrate formation. The inclusion of nanoparticles (with a particle size between 1 and 100 nm) not only offers heterogeneous nucleation sites, thereby reducing the activation energy barrier to nucleation, but also greatly enhances the heat transfer characteristics of the system [34]. Studies conducted by Alibadi et al. showed nanoparticles (CuO) significantly increased the kinetics of hydrate formation [35]. Ahuja demonstrated the promoting effect of hydrophobic nanosilica on hydrate growth when combined with a surfactant (Span 80) [36]. 

It is important to note that one limitation of using nanoparticles as kinetic hydrate promoters (KHPs) is the cost and difficulty associated with the synthesis process. Furthermore, nanoparticles have a high tendency to aggregate in the bulk phase due to their large surface area, thereby gradually losing their hydrate promoting effect over time, unless they are stabilized by surfactants. As a result, recent efforts have focused on improving the kinetics of hydrate formation through methods that enhance contact between the guest and the water molecules. A wide range of porous media has been investigated, including metal-organic frameworks (MOFs) [37], silica sands [38,39,40], silica gels [39,41], superabsorbent polymers (SAP) [42,43,44], glass beads [45], clay [46], activated carbon [47,48], nanotubes [49,50], and biological porous materials [51]. If we disregard the impact of porous media on the phase equilibrium of gas hydrates, it is commonly believed that such media can provide a greater number of nucleation sites [52,53], increase the gas-liquid contact areas [54], thus promoting hydrate formation kinetics. The presence of porous media can also dissipate the latent heat of hydrate formation quickly [55] and improve the heat transfer efficiency. Additionally, it has been reported that Succinic Acid-Polyethylene Glycol (SAP) possesses the ability to promote hydrate formation kinetics while retaining methane storage capacity even after undergoing up to 20 cycles of hydrate formation and dissociation under specific temperature and pressure conditions [43].

**Figure 1:**
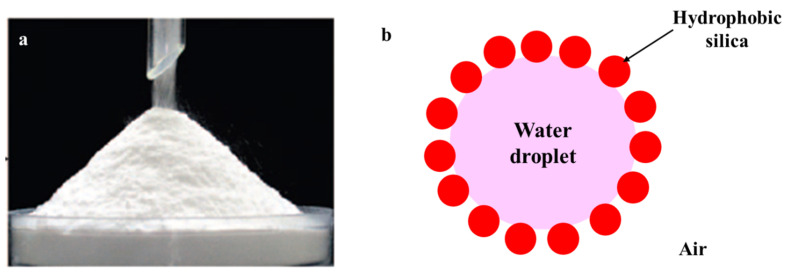
(**a**) Dry water prepared with 5 g of hydrophobic nanosilica and 95 g of water (with permission from Reference [58]); (**b**) mechanism of hydrophobic silica-stabilized dry water (water-in-air Pickering dispersion).

The impact of factors, such as water saturation, gas flow rate, particle size, and the morphology of porous media on CO_2_ hydrate and methane hydrate formation and dissociation processes, has been extensively investigated in References [39,40], respectively. It is disappointing that the particle size of porous media has been found to have an opposite effect on hydrate formation kinetics and gas storage capacity [56]. In recent years, a novel type of water-in-air Pickering dispersion (aerosol), known as dry water, has emerged as an effective promoter of hydrate formation. Dry water droplets are primarily composed of water and nanoparticles, with water being the primary component. While it was first developed in 1964, it was not until the 1990s that scientists began to extensively study its properties [57]. The application of dry water to hydrate promotion was firstly proposed by Wang et al. in 2008 [58]. As its name suggests, dry water has the appearance of a free-flowing powder. We note that, although dry water has an appearance of a powder, dry water is in fact a dispersion (aerosol) in which water is the dispersed phase (droplets) and air is the continuous phase. It is a particle-stabilized dispersion composed of approximately 95% (*w/w*) water and 5% (*w/w*) hydrophobic nanosilica (Pickering agent). Figure 1 depicts the appearance and stabilizing mechanism of dry water. 

As a promoter of hydrate formation, dry water droplets are capable of significantly increasing the contact area between the guest and the water molecules, thus promoting kinetics of gas hydrate formation [58,59]. Additionally, the structure of water molecules (not the molecular structure of H_2_O but the orientation of the molecules) can be reorganized in the vicinity of a hydrophobic surface [60], and the guest gas can be adsorbed at the surface of hydrophobic nanosilica to form a dense gas layer [61], thereby promoting hydrate formation thermodynamically. The preparation of dry water is straightforward, and it is non-toxic and environmentally friendly, making it a promising candidate for promoting hydrate formation. However, before dry water can be widely used, several major challenges still need to be addressed. This review collates the progresses made in dry water research and provides a reference for future studies exploring dry water as a promoter of hydrate formation. 

## 2. Formation of Stable Dry Water

### 2.1. Preparation Procedure of Dry Water

The materials used in the preparation of dry water consist of solid stabilizers, primarily hydrophobic nanosilica, water, and a high-speed blender. The preparation process involves high-speed shear blending, which is necessary to disperse both the nanosilica particles and bulk water simultaneously. This process ensures that the nanosilica particles adsorb at the water-air interface and stabilize the dispersion immediately, preventing the water droplets from agglomerating back into a bulk phase [62]. Table 1 provides a summary of the properties of dry water synthesized by various research groups. 

### 2.2. The Effect of Nanoparticle Size

It is reasonable to expect that the particle size of nanosilica would have an impact on the formation of dry water. Thermodynamically, the energy required to remove a nanoparticle from a water-air interface can be expressed by Equation (1):

(1) $$∆E=\pi R^{2}\gamma_{aw}{\left(1\pm cos\theta \right)}^{2},$$

where the R is the radius of the nanoparticle, γ_aw_ is water-air interfacial tension, and θ is the contact angle of water on the nanoparticle. Equation (1) shows that the size of nanoparticles has a direct impact on the energy required to remove them from a water-air interface into either the bulk water phase or to the bulk air phase. The negative sign in the bracket applies when removing the nanoparticle to the water phase, whereas the positive sign applies when removing it to the air phase. While a larger nanoparticle size is more thermodynamically favorable, it cannot be too large because dispersing them becomes more challenging as the particle size increases. Furthermore, a larger particle size results in a looser and more incomplete coating of the water surface by the particles, which increases the likelihood of water droplet aggregation. This is unfavorable for the stability of dry water. 

Previous studies reported that nanosilica particles with an average diameter of 20 nm were the most suitable for dry water preparation [62], although other studies reported successful dry water production with nanosilica particles of typical sizes ranging from 30 to 50 nm [84]. Additionally, Rong et al. discovered that silica nanoparticles with the same hydrophobicity but different sizes (350 nm vs. 15 nm) formed different products with water under identical blending conditions [62]. The larger nanoparticles formed a soufflé-like mixture, while the smaller ones formed dry water [62]. Furthermore, Rong et al. emphasized the importance of large pores in stabilizing dry water [62].

### 2.3. The Effect of Nanoparticle Shape

**Figure 2:**
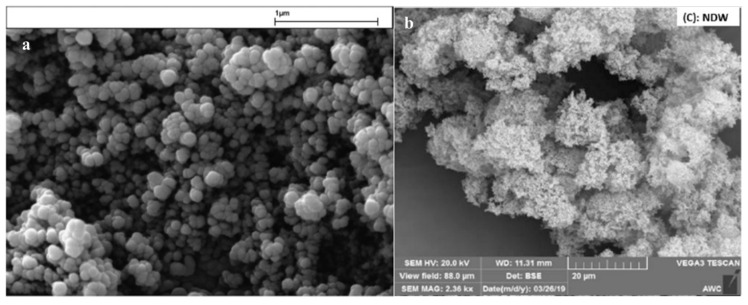
SEM image of HDK-H18 hydrophobic nanosilica and dry water droplet in the presence of 20 wt% Aerosil R812S hydrophobic nanosilica, reprinted from [73] and [82], respectively, with the permission of copyright.

Zhang et al. succeeded in producing dry water using spherically shaped nanosilica [84]. Other studies showed that the powdered hydrophobic HDK-H18 nanosilica showed a spherical shape under the scanning electron microscopy (SEM) [73]. Transmission electronic microscope (TEM) pictures showed the shape of Aerosil R812S and R 972 nanosilica in [63]. Figure 2 shows the SEM image of HDK-H18 hydrophobic nanosilica and dry water droplet in the presence of 20 wt% Aerosil R812S hydrophobic nanosilica, reprinted from [73] and [82], respectively. 

### 2.4. The Effect of Particle Hydrophobicity

**Figure 3:**
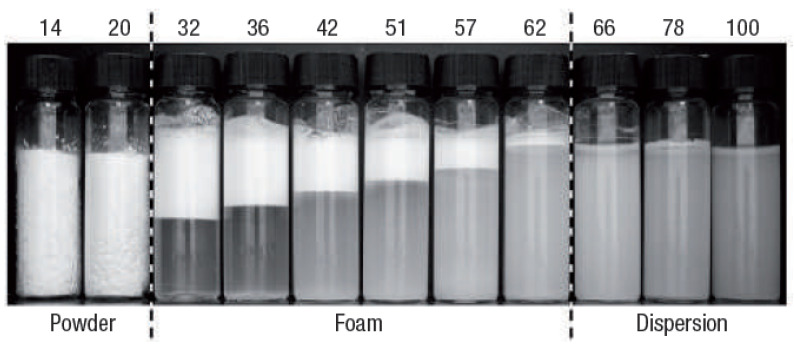
Phase transition of the final product after vigorous blending water with nanosilica from dispersion to dry water with the increase of hydrophobicity of nanosilica indicated by the decreased SiOH content showing at the top of the figure. Reprinted from [89] with permission.

The hydrophobicity of nanosilica is a crucial factor in determining the final product form after blending. For instance, Rong et al. prepared mesoporous nanosilica particles with a comparable size of 15 nm but different degrees of hydrophobicity [62]. Only nanosilica with deep hydrophobization treatment (water contact angle higher than 110°) of the particles with grain sizes ranging from 30 to 100 μm produced dry water after vigorous stirring with water. Less hydrophobic ones, on the other hand, formed a soufflé-like mixture. In another study, Forny et al. found that, as the hydrophobicity of nanosilica increased (from hydrophilic to superhydrophobic), the products after blending with water transitioned from suspension to mousse and eventually to dry water. However, only superhydrophobic nanosilica with a water contact angle of approximately 118° produced dry water [85]. Figure 3 shows the phase transition of the product after blending a water -nanosilica mixture, from dispersion to foam then to dry water, with increasing nanosilica hydrophobicity, characterized by the lower SiOH content indicated at the top of the figure. Without further surface modification by polydimethylsiloxy, which is used to prepare H18 nanosilica, the surface property of nanosilica is either hydrophilic or partially hydrophobic. Water has a non-zero contact angle on this naturally occurring nanosilica, and it can stabilize an air-in-water foam, in which the air is the dispersed phase and the water is the continuous phase. In addition to its influence on dry water stability, the hydrophobicity of nanosilica also affects size distribution of dry water droplet, with dry water prepared using more hydrophobic nanosilica tending to have a narrower and more uniform droplet size distribution [84].

Correlating the hydrophobicity of nanosilica with the product obtained after vigorous blending with water is a challenging task. One issue is the difficulty in quantifying the hydrophobicity of the nanoparticles, as even manufacturer instructions often only provide a qualitative evaluation without any quantitative characterization, such as the contact angle of water. Various methods have been proposed in the literature to quantify the hydrophobicity of nanoparticles, including the water intrusion method proposed by Forny et al. [57,85], the immersion microcalorimetric method proposed by Yan et al. [86] and Spagnolo et al. [87], the indirect calculation from surface free energy proposed by Elzer et al. [88], and the photogoniometric method proposed by Rong et al. [62]. These methods usually involve compressing the nanoparticles into a macro pellet before direct measurements or indirectly calculating the contact angle mathematically. Therefore, a direct and accurate method is required in the future to quantify the hydrophobicity of nanosilica. 

### 2.5. The Effect of Experimental Conditions

Han et al. investigated the effects of silica-to-water ratio and blending conditions on the stability of dry water using bis(trimethylsilyl)amine (HMDS)-treated hydrophobic silica particles. They found that an excess of water resulted in insufficient coating of hydrophobic silica on water droplet surface, while an excess of hydrophobic silica resulted in too many uncoated hydrophobic silica particles, leading to a waste of raw material [68]. Mixing time that is too long or too short is also not conducive to the stability of dry water. After optimization, they determined that 1 g of hydrophobic silica should be mixed with 95 g of water under 30 s of blending at a speed of 22,000 rpm. Other studies, such as Zhang et al., also reported suitable blending rates as 4000–6000 rpm [84], and Chen et al. [90] found 5000 rpm to be optimal. Zou et al. [67] identified a mass ratio of nanosilica to carrageenan solution of 9:100 at a mixing speed of 5000 rpm for 300 s as the best conditions for preparing dry water. Forney et al. [63] noted that a minimum mixing speed of 12,000 rpm is required for successful dry water formation, and the stability of the final product is sensitive to the energy input of high-speed blending. F. Farhang et al. [64] suggested that a turbulent flow and a mixing speed of at least 3000 rpm were required for the encapsulation of water droplets by nanosilica particles, a finding also noted by Saleh et al. [57]. Additionally, Saleh et al. [57] found that the impeller of the blender can also affect dry water formation efficiency, with a helical-shaped impeller being estimated to be the most efficient.

### 2.6. The Effect of the Aqueous Phase

The interfacial tension between water and air also plays a role in the stabilization of dry water [57], in addition to the hydrophobicity of the nanoparticles. The surface tension of water can decrease with heating, which has been observed to induce a transition of the final product from dry water to mousse [68,84,91]. B.P. Binks et al. [73] investigated the effect of surfactants on dry water formation and found that the final product may experience a phase inversion from dry water to foam when a surfactant like sodium dodecyl sulfate (SDS) was added to the aqueous solution. B.P. Binks et al. [74] also noted that a neutral/weakly basic environment or a high salt concentration favored the formation of dry water due to the hydrophobicity of colloidal particles. Additionally, Zhang et al. [84] found that dry water can be formed using inorganic salt solutions instead of pure water, in combination with hydrophobicized nanosilica particles, which is promising for potential applications in seawater desalination. 

### 2.7. Lifetime of the Dry Water 

Dry water remains stable for a long time without external perturbation, and increasing the hydrophobicity of the silica particles is beneficial for maintaining the water content at the initial mass ratio of water [92,93]. Research shows that the water content in a dry water sample only drops by 0.06–1.74% and 0.32–3.02% after storage of 3 and 6 months [84], respectively. In addition, adding a gel such as gellan gum can help strengthen the structure of dry water and prevent water loss over time [59,68,90,94]. The double helix structure formed in the presence of gellan gum has been noted by Carter et al. [59,77] as being responsible for this effect. 

### 2.8. The Effect of External Stresses on the Stability of Dry Water

The stability of dry water under high pressure and vigorous stirring is an important consideration for its practical applications for clathrate hydrate formation. It has been observed that under these external stresses, water is squeezed out from dry water, resulting in phase separation and instability. Data showed that dry water stabilized by nanoparticles and gel had a higher capacity to resist water loss under high pressures, compared to the dry water stabilized solely by nanoparticles [67,84]. Cooling the dry water droplet down to 270 K has no effect on its structure, but high pressures and stirring cause instability [64]. Higher silica-to-water ratios also help dry water resist loss of stability caused by high pressure and stirring because smaller water droplet prepared under high silica content better maintains original shapes [61]. Smaller dry water droplet size, prepared from a high silica-to-water ratio, also improves dry water stability under severe external stresses [64]. Therefore, it is important to consider these external factors when examining the stability of dry water.

In addition to high pressure and stirring, pH also impacts the stability of dry water. Rong et al. found that dry water stabilized by hydrophobic nanosilica underwent disassembly upon an addition of an acid due to the protonation of amine groups on the coating surface of silica particles [62]. B.P. Binks et al. found that only water with a pH between 7 and 9 forms dry water after blending with hydrophobic nanosilica. Therefore, pH is an important factor to consider when stabilizing dry water with hydrophobic nanosilica [74]. This point make CO_2_ hydrate formation in dry water might not be as stable as methane formation in dry water because the dissolution of CO_2_ in water renders the pH of the aqueous solution below 7.

### 2.9. Size Distribution of Dry Water Droplets

B.P. Binks et al. observed that the water droplet dispersed by nanosilica was non-spherical and had a size of several hundred micrometers [73]. The size of the water droplet decreased to 100 μm with an increase in surfactant (SDS) concentration up to 5 mM. Zou et al. reported that the size of dry water droplets is mostly between 150 and 270 μm [67]. The addition of a gel stabilizer led to the formation of dry water droplets with a more uniform size distribution compared to normal dry water in the absence of a gel [84]. The size of dry water droplets was influenced by the silica-to-water ratio [64,95] and the hydrophobicity of hydrophobic nanosilica [84]. It was found that a lower silica-to-water ratio (2 and 3 wt%) led to a larger average size of dry water droplets than a higher ratio (5 and 6 wt%) [64]. Similarly, other studies [66,78,96] also concluded that the dry water droplet size decreased with an increase in hydrophobic nanosilica concentration. The presence of a surfactant, such as SDS, also led to a decrease in dry water droplet size [73]. 

### 2.10. Alternative Material to Nanosilica

From the analysis presented above, we can conclude that the key factor for stabilizing water-in-air dispersion with a Pickering agent is its hydrophobicity. This suggests that alternative, more cost-effective materials could be used for the preparation of dry water. For example, Teflon powder has been reported as a successful stabilizer for dry water [80], and superhydrophobic candle soot [97] could also be a promising candidate due to its low cost and abundance. Additionally, after hydrophobic coating, nanocarbon soot and nanoclay are potential alternatives for stabilizing dry water. 

## 3. The Effect of Dry Water on Gas Hydrate Formation

### 3.1. Thermodynamic Effect

**Figure 4:**
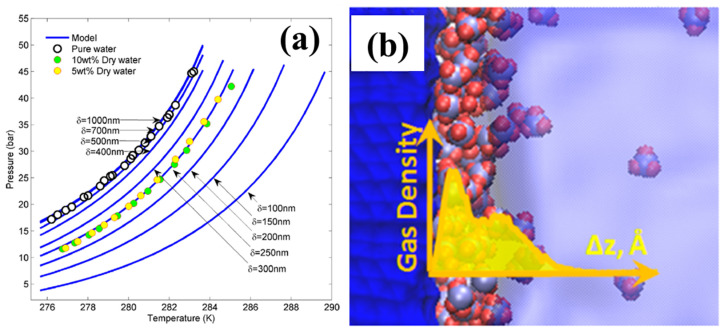
(**a**) Thermodynamic promoting effect of dry water containing different weight percent of hydrophobic nanosilica on CO_2_ hydrate equilibrium conditions, which was shifted to lower pressure region at a fixed temperature. Reprinted from [99] with permission. Circular symbols were equilibrium conditions determined experimentally, and solid lines represent the phase boundary of CO_2_ hydrate predicted by the thermodynamic model suggested by Zebardast et al. at different distances from the hydrophobic surface, δ. (**b**) Local gas enrichment at a hydrophobic surface-water interface obtained from molecular dynamic simulation, reprinted from [100] with the permission. ∆z is the distance measured from the interface.

Conventionally, the effect of dry water droplets on hydrate formation has been explained through a kinetic route. However, recent research suggested that dry water has a thermodynamic promoting effect on hydrate formation. Park et al. found that the equilibrium conditions for methane hydrate formation in dry water were shifted to higher temperatures and lower pressures compared to bulk water, indicating a thermodynamic effect on promoting hydrate formation [79]. Zebardast et al. confirmed the thermodynamic promotion of dry water on CO_2_ hydrate formation experimentally, utilizing a high-pressure stainless steel reactor by intersecting the heating and cooling curves of samples, as previously proposed by Tohidi et al. [98], and attributed it to the hydrophobic attraction force [99], as shown in Figure 4a.

Nguyen et al. proposed that the thermodynamic promoting effect was due to the hydrophobic effect. Water molecules became more ordered and clathrate-favorable near a hydrophobic surface, and the local gas concentration increased, which enhanced the formation of hydrates [100,101]. Farhang et al. suggested that the formation of a dense gas layer at the hydrophobic nanosilica-water interface provided nucleation sites for hydrate formation, as indicated in Figure 4b [61].

Li et al. used Raman spectroscopy to observe that water molecules were more ordered in the vicinity of a hydrophobic surface than in bulk water [60]. They also observed preferential gas hydrate formation on a hydrophobic surface in a hydrate formation experiment [53,101]. Molecular dynamics simulations showed local gas enrichment at the hydrophobic surface-water interface compared to bulk water. The thermodynamic promoting effect of dry water on CO_2_ hydrate equilibrium conditions was also demonstrated. 

### 3.2. Kinetic Effect

Zhang et al. investigated the use of dry water hydrate to separate and recover methane from coal mine gas. The researchers discovered that adding dry water not only enhanced the formation rate of methane hydrate, but also improved the purification of methane from raw gas, more so than when hydrate formation was promoted by stirring or other promoters [72]. In a separate study, Hu et al. measured the hydration number of dry water methane hydrate (6.224 ± 0.175) and confirmed its homogeneity using Raman spectra [81,102]. Wang et al. also reported that the presence of dry water reduced the induction time of methane hydrate nucleation to 5–10 min under quiescent conditions [58]. Additionally, Drachuk et al. observed that an absence of an induction time for propane hydrate formation in frozen dry water compared to the typically long induction time for hydrocarbon gas hydrate [65]. This promoting effect occurred due to the enhanced guest-water contact and therefore more efficient methane diffusion into water, compared to methane hydrate formation at the methane-bulk water interface. This mechanism is consistent with the finding that hydrate formation rate in dry water closely correlated with the water droplet size dispersed by hydrophobic nanosilica [58]. 

**Figure 5:**
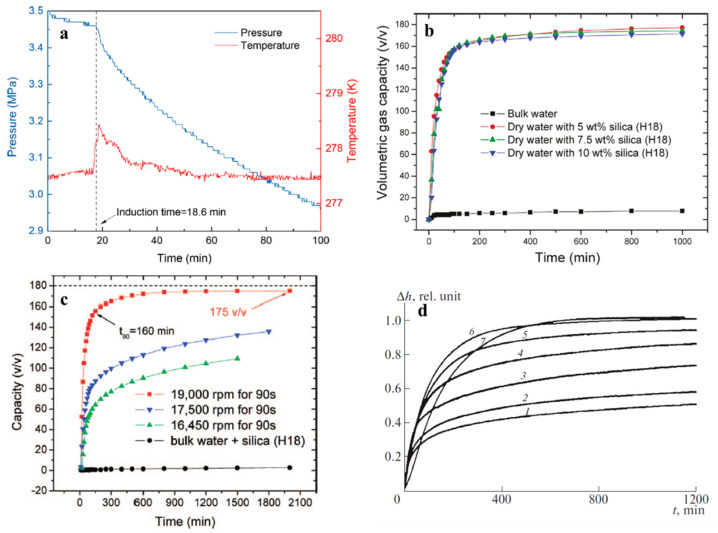
Representation of the promoting effect of dry water on clathrate hydrate formation parameters. (**a**) The induction time of CO_2_ hydrate was shortened to 18.6 min in the presence of dry water, as indicated by the pressure down and temperature up in the profile. Reprinted from [104] with permission. (**b**,**c**) Gas uptake by formation of clathrate hydrate as a function of time. Compared with bulk water, dry water droplets containing different silica content and prepared by different mixing speed showed consistently higher gas storage capacity, despite the large effect of mixing speed on it. Reprinted from [59] and [58] with permission, respectively. (**d**) Water-to-methane hydrate conversion as a function of time. The number aside each curve indicated the silica content present in dry water: (1) 2 wt%, (2) 3 wt%, (3) 5 wt%, (4) 7 wt%, (5) 10 wt%, (6) 12 wt%, and (7) 15 wt%. It can be seen that water-to-hydrate conversion increased with the increase of silica content and approached 100% at highest silica concentration (15 wt%). Reprinted from [75] with permission.

Figure 5 shows the kinetic promoting effect of dry water on clathrate hydrate formation. The induction time of methane hydrate was shortened to 18.6 min in the presence of dry water, and compared to the bulk water system, the gas storage capacity of the dry water clathrate hydrate exhibited a surge. Another kinetic parameter, water-to-hydrate conversion, defined as the ratio of the mass of water converted to hydrate crystal to the initial mass of water, also increased with the presence of nanosilica in dry water. As discussed above, H18 nanosilica stabilized water droplets with a size in the tens of micrometers. To further decrease the droplet size, one could increase the mixing speed, but this requires higher energy input and may not be suitable for the large-scale production of dry water. Alternatively, a superhydrophobic nanomaterial could be synthesized to prepare dry water with a narrower size, thus enhancing the hydrate formation rate. In addition to the mass transfer problem, another obstacle that hinders fast hydrate formation is the heat transfer issue. Fan et al. proposed a solution to this problem by combining frozen dry water with heat-conducting nanoparticles (nanocopper) to improve heat exchange and dissipate the heat released by hydrate formation faster [103].

Wang et al. conducted a study on CO_2_ hydrate formation kinetics in dry water stabilized by Teflon particles of various sizes [80]. The presence of dry water was found to significantly decrease the induction time of CO_2_ hydrate formation, as a result of the increased mass transfer across the gas-water interface. This reduction depended on both the size of the Teflon particle and its weight percent in dry water. Farhang et al. also reported that CO_2_ hydrate formation kinetics was greatly enhanced by dry water, with the degree of enhancement dependent on the mass concentration of nanosilica present [61]. Compared to pure water systems, the presence of dry water led to a marked increase in CO_2_ gas consumption, maximum CO_2_ uptake, and CO_2_-to-hydrate conversion. The induction time was also significantly shortened to as little as 10 min, indicating a prominent promoting effect of dry water on CO_2_ hydrate formation. Zhang et al. confirmed these findings, observing that the increase in nanosilica content in dry water droplets led to a decrease in induction time, an increase in gas uptake, and an increase in water-to-hydrate conversion of CO_2_ hydrate [104]. Based on the shrinking core model, they calculated the effective gas diffusion coefficient through the hydrate shell and found that the impact of the latent heat released by hydrate formation on the formation kinetics was negligible. B.O. Carter et al. investigated the effect of dry water on the formation kinetics of methane hydrate (structure I), carbon dioxide hydrate (structure I), and krypton hydrate (structure II) [59]. Their results showed that the gas uptake kinetics of all three types of hydrates were improved, indicating that the promoting effect of dry water is not limited to the crystal structure of clathrate hydrates. 

### 3.3. Enhancement of the Gas Storage

The literature shows that the gas storage capacity of clathrate hydrate formation is significantly enhanced in the presence of dry water. Methane uptake capacity is orders of magnitude higher in dry water methane hydrate than in methane hydrate formed at the methane-bulk water interface [58,59]. The amount of methane stored in dry water methane hydrate depends on water droplet size [58] and temperature [59,79], with the highest values observed at a mixing speed of 19,000 rpm [58] and a hydrate formation temperature of 273–277 K [79]. The mixing speed used for the preparation of dry water has a greater impact on the gas uptake kinetics of dry water methane hydrate than the silica-to-water ratio [59,79]. Dry water methane hydrate exhibits increased gas storage capacity compared to methane hydrate formation in the presence of a surfactant aqueous solution, although it is slightly influenced by the mixing speed used for dry water preparation [81]. While large cages of dry water methane hydrate are almost fully occupied, small cages remain approximately 10% vacant [81], indicating that future work could focus on enhancing small cage occupancy to further improve methane storage capacity using dry water clathrate hydrate. 

### 3.4. Synergies with Other Chemicals

The synergistic effect of dry water and other hydrate promoters is a topic that is worth exploring, with surfactants being known as effective kinetic promoters for hydrates of different guest types [12,13,14,105]. Typically, kinetic parameters such as gas uptake capacity, gas uptake rate, water (or guest) conversion to hydrate, nucleation rate, and growth rate of hydrate crystals can be utilized to measure the synergistic effect between dry water and other additives. In one study, Fan et al. performed hydrate formation experiments in a dry solution of sodium dodecyl sulfate and compared the gas storage kinetics of methane hydrate in dry surfactant solution with that of dry water methane hydrate [78]. Both hydrate formation in dry surfactant solution and dry water greatly enhanced methane hydrate formation rates compared to bulk water systems, and the former was found to be more effective. It was also found that methane hydrate in dry surfactant solution exhibited the same final methane storage capacity (around 170 m^3^/m^3^) as dry water methane hydrate but with faster storage rates, as confirmed by 60 min of t_90_ (the time taken to achieve 90% of final gas uptake) for dry surfactant solution and 200 min of t_90_ for dry water. The authors attributed the superior gas uptake kinetics of methane hydrate in dry surfactant solution to the better dispersion of water by dry water and the lower activity of water due to the presence of sodium dodecyl sulfate molecules.

On the other hand, Farhang et al. reported no synergistic effect (CO_2_ to hydrate conversion kept in between 40 and 50% for both dry water and THF + dry water systems) between dry water and the thermodynamic hydrate promoter (THP) tetrahydrofuran (THF) for any of the mixture ratios on CO_2_ hydrate formation kinetics [61]. 

To date, there have been no reports on the synergistic effect of dry water and other additives on the nucleation of gas hydrates due to the challenges associated with determining nucleation rate. This has been one of the research topics in our group. Our current research involves investigating the impact of seven ice nucleation promoters [106] on the kinetics of CO_2_ hydrate formation. 

### 3.5. Comparison to Other Hydrate Promoters

Studies have compared the promoting effects of dry water and surfactants, such as sodium dodecyl sulfate (SDS) and other promoters, on gas hydrate formation and gas storage kinetics. The results indicate that SDS is more effective on enhancing hydrate formation kinetics than dry water [59], while dry water hydrate exhibits a higher gas storage capacity than hydrate formed in the presence of SDS [59,81]. The performance of dry water has also been found to be comparable to thermodynamic hydrate promoter (THP) such as THF [61]. These findings highlight the potential of combining dry water with other promoters to achieve even greater improvements in gas storage capacity and kinetics. 

## 4. The Effect of Dry Water on the Self-Preservation Effect

**Figure 6:**
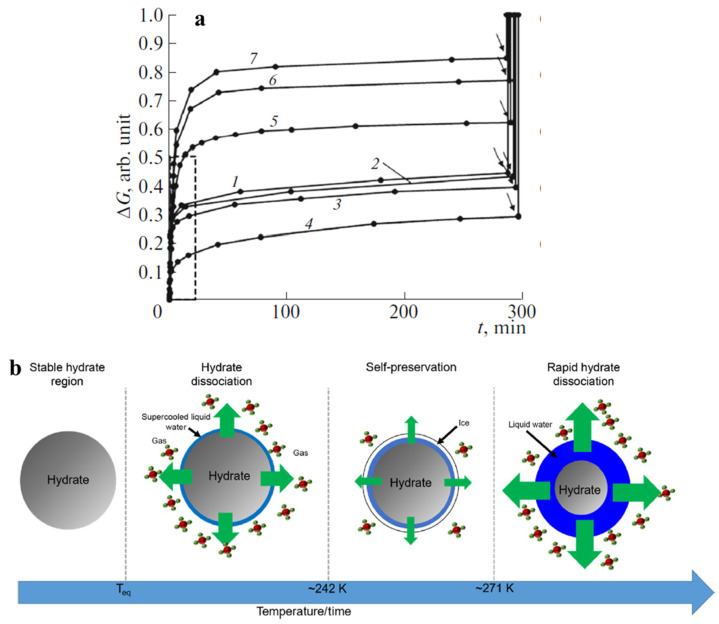
(**a**) Methane hydrate dissociation profile at 268.2 K and 0.1 MPa, where ∆G represents the degree of dissociation of methane hydrate. The nanosilica content in dry water (wt%) is 2% (curve 1), 3% (curve 2), 5% (curve 3), 7% (curve 4), 10% (curve 5), 12% (curve 6), and 15% (curve 7). It can be found that, before starting heating the system above 273 K (the point of the arrow marker), methane hydrate kept an anomalous low rate of dissociation, which is the so called “self-preservation” effect. Reprinted from [75] with permission. (**b**) The proposed mechanism of the self-preservation effect. Within hydrate metastability region below ice melting point, if the free water was in the state of supercooled liquid phase, rapid hydrate dissociation occurred, while if the free water was in the state of ice, the self-preservation effect manifested and the hydrate dissociated at a slow rate because the ice crust coating on the surface of hydrate particle hindered the dissociation of hydrate and the release of guest gas to surroundings. Reprinted from [109] with permission.

The use of clathrate hydrates for gas transportation requires not only fast formation kinetics, but also low pressures for safety and cost efficiency. The self-preservation effect, which is the anomalous slow dissociation kinetics and retarded gas release at atmospheric pressure and temperatures below 273 K, has been observed even though these conditions are outside the thermodynamic stability zone of the gas hydrate phase diagram [65,81,96]. This effect renders gas hydrates suitable for gas storage and transportation [107,108]. Figure 6 illustrates the dissociation profile of methane hydrate obtained in dry water containing different amounts of nanoparticle stabilizer (R202) at 268.2 K and 0.1 MPa (top panel), and the proposed mechanism for the self-preservation effect (bottom panel). It is believed that the self-preservation effect is caused by ice formation on the surface of the hydrate particle during its early dissociation stages, which hinders gas release from hydrates. 

Although the self-preservation effect of dry water methane hydrate was observed by Hu et al., with a slowed-down dissociation rate, 55% of stored gas had been released before the effect occurred [81]. Therefore, it is important to continue investigating the self-preservation effect to enhance gas storage and transportation capabilities of dry water clathrate hydrates. 

According to Melnikov, dry water methane hydrate exhibits a self-preservation effect, which increases with the size of water droplets [96]. Drachuk and Melnikov discovered that the efficiency of this effect depended on the phase state of unreacted water, with the hydrate remaining metastable without dissociation when residual water was in the supercooled liquid state and pressure was below the hydrate equilibrium pressure [65,110,111,112]. The self-preservation effect was also dependent on the size of the hydrate particle, and was only observed for hydrate particles larger than 250 μm [96]. As water droplet size increased, methane hydrate particles had a higher bulk fraction, which was conducive to the self-preservation effect but not to gas storage and hydrate formation rate. Podenko et al. studied the effect of nanosilica content on methane hydrate formation rate and the self-preservation effect and found that dry water with 7 wt% of R202 hydrophobic nanosilica had the optimal balance between the high hydrate formation rate and the high efficiency of self-preservation effect [75].

## 5. The Reusability of the Dry Water

The reusability of dry water has been addressed through several studies. B.O. Carter et al. developed a dry gel by blending a 3 wt% aqueous solution of gellan gum or gellan gum gel with hydrophobic nanosilica. The dry gel showed improved reusability, maintaining its properties for at least four successive hydrate formation-dissociation cycles without re-blending [59]. Similarly, Yang et al. investigated the stabilization of dry water methane hydrate particles through the addition of gellan gum powder [113]. Although the stabilized dry water could be reused for nine cycles, the induction time became longer and the gas uptake capacity showed attenuation after three cycles, indicating that the addition of gel could only mitigate destabilization to a limited extent. 

Golkhou et al. extended the study to CO_2_ hydrate and showed that the addition of 15 wt% gelling agent exhibited better recyclability over seven consecutive hydrate formation-dissociation cycles [114]. However, it should be noted that the improvement of reusability came at the cost of hydrate formation and gas storage kinetics due to the larger droplet size of the dry gel compared to that of dry water. Park et al. claimed that dry water gradually lost its stability, leading to a decrease in the hydrate fraction with the hydrate formation-dissociation cycles because of the phase separation of free water from dry water [79], which was confirmed using Raman spectra by Hu et al. [102]. 

**Figure 7:**
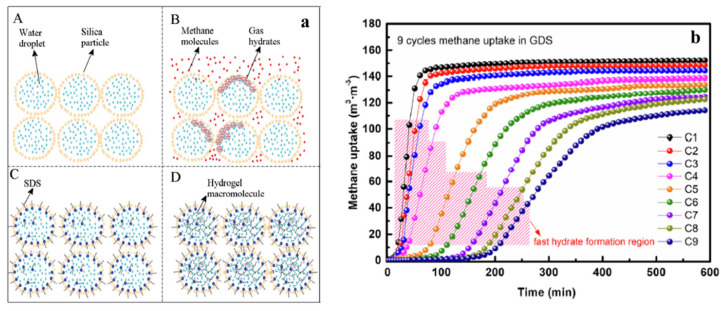
(**a**) The schematic diagram of pure dry water (**A**), dry water methane hydrate (**B**), dry surfactant solution (**C**) and dry gel (**D**). Copied from [116] with the permission of He et al. (**b**) Gas uptake capacity of methane dry water hydrate with time during up to nine hydrate formation-dissociation cycles. It was shown that with the increased number of reuses of dry water, methane uptake not only showed a decreased amount, but also showed a slowed rate. Copied from [113] with the permission of Yang et al.

Ding et al. and Shi et al. investigated the effect of different superabsorbent hydrogels on the recyclability of dry water after hydrate dissociation [69,115]. Despite having slightly inferior gas storage capacity, the addition of superabsorbent hydrogel significantly improved the recyclability of dry water, which could be reused for at least eight cycles [69]. The increased hydrate formation kinetic parameter and recyclability of dry water in the presence of porous hydrogel particles were quantitatively analyzed using the shrinking-core model [115]. Although the recyclability of dry water was improved in the presence of hydrogel, the relationship between hydrate crystal size and the pore size of the hydrogel network has not been established so far, which may be a potential subject of future research direction. Figure 7 illustrates the schematic diagram of pure dry water, dry water methane hydrate, dry surfactant solution, and dry gel (left panel), as well as the methane uptake capacity in gelatinous dry solution (GDS) during nine hydrate formation-dissociation cycles (right panel).

## 6. Future Research Directions

Dry water has shown promise as a potential gas storage medium using clathrate hydrate formation, but there are still several challenges that need to be addressed. Firstly, the preparation of dry water using hydrophobic nanosilica requires a significant amount of energy input, making it not cost-effective for large-scale production. Additionally, alternative Pickering agents that can disperse water through self-assembly need to be explored to replace hydrophobic nanosilica. Secondly, while dry water has proven to be effective in promoting CO_2_ and methane hydrate formation kinetics and enhancing respective gas storage capacity, there is still a gap in research on hydrogen storage in the form of dry water hydrate. The primary objective in hydrogen hydrate formation is to decrease the formation pressure. One approach is to explore the use of thermodynamic hydrate promoters, such as cyclopentane, which can form mixed gas hydrates with hydrogen at pressures considerably lower than that required for pure hydrogen hydrate formation. Additionally, although it is evident that the nanosilica content in dry water dispersion impacts gas hydrate formation kinetics by forming water droplets of various sizes, the effect of the shape of nanosilica on gas hydrate formation kinetics remains inadequately understood and could be a fascinating subject for future research.

Fourthly, the reusability of dry water over hydrate formation-dissociation cycles without re-blending is a problem that still needs to be solved. Even though the addition of gelling agents has improved stability, the destabilization of dry water still occurs with increasing cycles. In the presence of hydrogel, the effect of hydrogel on the morphology of dry water clathrate hydrate also needs to be characterized. Fifthly, the self-preservation effect of dry water hydrate has not been thoroughly investigated, and maximizing its effectiveness during transportation of gas-stored dry water hydrate of different hydrate formers requires further research.

## 7. Conclusions

The high gas uptake capacity of clathrate hydrate makes it an attractive material for gas storage and transportation, particularly for methane, carbon dioxide, and hydrogen. Gas storage in clathrate form is also much safer than traditional methods such as liquefaction or compression. However, slow formation kinetics due to mass transfer limits have hindered large-scale applications of clathrate hydrate for gas storage and transportation. While many methods have been applied to enhance formation kinetics, such as spraying, stirring, and adding hydrate promoters, these have limitations for large-scale applications. Recently, dry water, a water-in-air dispersion stabilized by hydrophobic nanoparticles, has shown promising results in enhancing gas hydrate formation kinetics and gas storage capacity. This review summarizes the stabilizing mechanism of dry water droplet and the effect of physical conditions encountered during hydrate formation on the stability of dry water. The promoting mechanism of dry water on hydrate formation is also explored, with representative hypotheses listed. Extensive studies have shown that dry water has an excellent promoting effect on hydrate formation kinetics and gas storage capacity in the form of clathrate hydrate. However, existing critical issues, such as the destabilization of dry water after several formation-dissociation cycles, need to be addressed to enable large-scale applications of dry water in enhancing gas hydrate formation kinetics. This review also discusses future research directions for dry water hydrates.

## Figures and Tables

**Table 1:** Dry water properties in open literature.

Pickering Agent Name	Pickering Agent Size	Solid-to-Water Ratio	Mixing Speed and Time	Dry Water Droplet Size	Reference
Aerosil R812S nanosilica	7 nm	4:96	>12,000 rpm, 30 s	d_50_ = 112 μm	[63]
Aerosil R972 nanosilica	16 nm	10:90	18,000 rpm, 10 s	d_50_ = 131 μm	[63]
Wacker H18 nanosilica	5–30 nm	2–6 wt%	14,000 rpm, 180 s	d_50_ = 151–191μm *	[64]
Aerosil R202 nanosilica	14 nm	5–10 wt%	18,700 rpm, 60 s	d_50_ = 4 μm	[65,66]
Aerosil R812S nanosilica	7 nm	9:100	5000 rpm, 300 s	50 μm < d_50_ < 100 μm	[67]
Mesoporous Silica Particles	14 nm	5 wt%	1800 rpm, 180 s	30 μm–100 μm	[62]
HB-630 nanosilica	5–15 nm	5 wt%	22,000 rpm, 30 s	N/A	[68]
HB-630 nanosilica	5–15 nm	1:17	18,000 rpm, 45 s	15 μm	[69,70]
Wacker H18 nanosilica	7–35 nm	7.5 wt% **	18,000 rpm, 60 s	25–50 μm	[71]
HB-630 nanosilica	5–15 nm	5 wt%	19,000 rpm, 90 s	N/A	[72]
Wacker H18 nanosilica	20 nm	4 wt%	25,000 rpm, 30 s	several hundred μm ***	[73]
Wacker H18 nanosilica	20–30 nm	2 wt%	25,000 rpm, 30 s	50–several hundred μm	[74]
Aerosil R202 nanosilica	14 nm	2–15 wt%	19,000 rpm, 90 s	6–16 μm *	[75]
Wacker H18 nanosilica	20 nm	5 wt%	19,000 rpm, 90 s	<20 μm	[58]
Wacker H18 nanosilica	5–30 nm	10 wt%	16,450 rpm, 60 s	N/A	[76]
Wacker H18 nanosilica	5–30 nm	5 wt%	14,000 rpm, 180 s	100–5500 μm	[61]
Wacker H18 nanosilica	5–30 nm	5 wt%	19,000 rpm, 90 s	52 ± 14 μm	[59]
Wacker H18 nanosilica	5–30 nm	5 wt%	37,000 rpm, 90 s	26 ± 17 μm	[77]
HB-630 nanosilica	5–15 nm	2.5–10 wt%	18,000 rpm, 30 s	1–120 μm *	[78]
Wacker H18 nanosilica	5–30 nm	5 wt%	19,000 rpm, 60 s	13 μm	[79]
Teflon particle	1 and 12 μm	15 wt%	14,100 rpm, 180 s	N/A	[80]
Wacker H18 nanosilica	5–30 nm	5 wt%	12,000, 17,000, 22,000 rpm, 90 s	N/A	[81]
Aerosil R812	7 nm	20 wt%	14,000 rpm, 10 s	N/A	[82]
HB-630 nanosilica	5–15 nm	5–8 wt%	17,000 rpm, 25 s	100 μm < d_50_ < 300 μm *	[83]

d_50_: median droplet size. * The median droplet size depends on the silica-to-water ratio; ** This ratio is the weight of nanosilica to the total weight of the mixture of SDS solution and gel; *** Dry water droplet size depends on the molarity of surfactant solution. N/A: relevant information not provided in the literature.

## Data Availability

Not applicable.
